# Miniaturized pH Sensors Based on Zinc Oxide Nanotubes/Nanorods

**DOI:** 10.3390/s91108911

**Published:** 2009-11-09

**Authors:** Alimujiang Fulati, Syed M.Usman Ali, Muhammad Riaz, Gul Amin, Omer Nur, Magnus Willander

**Affiliations:** Department of Science and Technology (ITN), Campus Norrköping, Linköping University, SE-60174 Norrköping, Sweden; E-Mails: syeal@itn.liu.se (S.A.); riamu@itn.liu.se (M.R.); gulam@itn.liu.se (G.A.); omeno@itn.liu.se (O.N.); magwi@itn.liu.se (M.W.)

**Keywords:** ZnO nanotubes, ZnO nanorods, pH sensors, potentiometric measurements

## Abstract

ZnO nanotubes and nanorods grown on gold thin film were used to create pH sensor devices. The developed ZnO nanotube and nanorod pH sensors display good reproducibility, repeatability and long-term stability and exhibit a pH-dependent electrochemical potential difference versus an Ag/AgCl reference electrode over a large dynamic pH range. We found the ZnO nanotubes provide sensitivity as high as twice that of the ZnO nanorods, which can be ascribed to the fact that small dimensional ZnO nanotubes have a higher level of surface and subsurface oxygen vacancies and provide a larger effective surface area with higher surface-to-volume ratio as compared to ZnO nanorods, thus affording the ZnO nanotube pH sensor a higher sensitivity. Experimental results indicate ZnO nanotubes can be used in pH sensor applications with improved performance. Moreover, the ZnO nanotube arrays may find potential application as a novel material for measurements of intracellular biochemical species within single living cells.

## 1. Introduction

ZnO is a promising material due to the wide direct band gap (3.37 eV) and large exciton binding energy (60 meV). Recent studies have shown considerable attraction towards ZnO nanostructures, particularly on one-dimensional ZnO nanorods and nanowires due to the fact that, for a large number of applications, shape and size of the ZnO nanostructures play a key role for the performance of the devices. Thus ZnO nanorods and nanowires have a variety of application in the field of optoelectronics [1,2], nanomechanics [3,4], nanosensors [5-11], resonators [12], electric nanogenerator [13], nanolasers [14] and a variety of methods are used to grow these [15-17].

pH determination is a strong prerequisite for many biochemical and biological processes. The use of ZnO nanorods and nanowires for pH sensing and miniaturization of pH sensors have attracted considerable interest since the large surface-to-volume ratio leads to a short diffusion distance of the analyte towards the electrode surface, resulting in an improved signal-to-noise ratio, faster response times, enhanced analytical performance, and increased sensitivity [18,19]. Recently we have also reported the successful demonstration of the use of ZnO nanorods to measure the intracellular pH in human fat cells [20], which also proves that ZnO nanostructures have unique biological advantages including non-toxicity, bio-safety, bio-compatibility and high electron communication features and make them one of the most promising materials for biosensor application.

As compared to ZnO nanorods and nanowires, ZnO nanotube structures possesses lots of interesting unique properties such as porous structures and large surface areas. Recently there have been reports on the use of ZnO tubular structures as sensors with improved performance and higher sensitivity compared to ZnO nanorods and nanowires [21-23]. However, no report has appeared yet of the use of ZnO nanotubes as pH sensors.

In this study, we report the fabrication of newly developed ZnO nanotube pH sensor by a two-step method (low-temperature aqueous chemical growth (ACG) of well aligned ZnO nanorods followed by etching ZnO nanorods to get ZnO nanotubes) and its comparison to a ZnO nanorod pH sensor. Our results show a linear response of the electrochemical potential of the developed pH sensor to various pH values and as high as twice the sensitivity of the ZnO nanorod pH sensor. This shows the great potential in using ZnO nanotubes for pH sensing with improved performance.

## 2. Experimental

### 2.1. Sample Preparation

**Figure 1.**
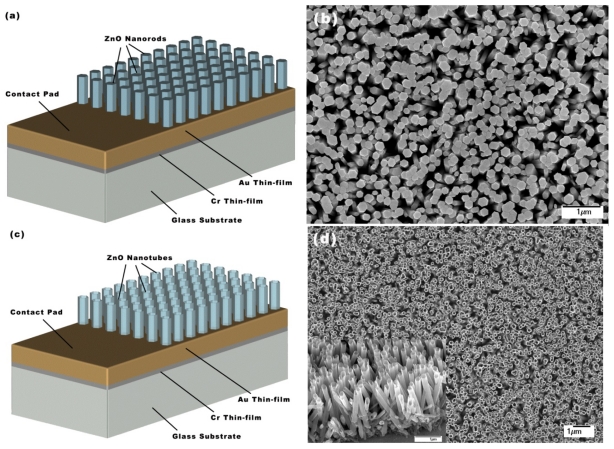
(a) and (c) schematic diagram of ZnO nanorod and nanotube pH sensors, respectively; (b) and (d) SEM images of ZnO nanorods and nanotubes, respectively (insert in image (d) shows tilted cross sectional view of nanotubes. The scale bar is 1 μm).

For all the developed pH sensors, glass was used as a substrate after being cleaned with acetone, de-ionized water and isopropanol. A chromium (Cr) thin film with 25 nm thickness was evaporated as an adhesive layer then a gold (Au) thin film with 100 nm thickness was evaporated as a gold electrode. The vertically well-aligned hexagonal ZnO nanorods were then grown on top of the gold thin film for 3–5 h using a low temperature method described in [24-26]. A small part of the glass substrate was covered during growth and was used as a contact area, as shown in Figure 1(a). In the ACG method, zinc nitride hexahydrate [(Zn(NO_3_)_2_6H_2_O)] was mixed with hexamethylenetetramine [C_6_H_12_N_4_] using the same molar concentration for both solutions. The molar concentration was varied from 0.025 M to 0.075 M. The two solutions were stirred together and the substrates were placed inside the solution. Then, it was heated up to 90 °C for 3–5 h. After the growth was completed, the samples were cleaned in de-ionized water and left to dry in air inside a closed beaker.

We have previously reported that sensitivity of ZnO nanorod pH sensor increases with the reduction in size of the nanorods [27]. Thus it is very crucial to get the same dimensions of ZnO nanotubes and nanorods (same density, uniformity, length and diameter of the ZnO nanotubes and nanorods) in order to accurately compare the sensitivity of ZnO nanotube and nanorod pH sensors.

In the second step, in order to get the same dimensions of ZnO nanotubes, we took some electrodes of previously obtained ZnO nanorods and after performing carefully chemical etching of ZnO nanorods along the c-axis direction described in [28], we finally obtained the required same dimension nanotubes. ZnO nanotubes were obtained by etching the as grown ZnO nanorods. After the growth of ZnO nanorods arrays, we divide the sample into two pieces. One piece was immersed in KCl solution of a concentration in the range from 0.1 M to 3.4 M for time periods ranging from 3 to 17 h to obtain the ZnO nanotube arrays. The temperature of the solution was kept at 95 °C. After 17 h of the immersion time in 3.0*M* KCl solution at 95 °C, we get tubular form of ZnO nanotube arrays with good yield.

A typical SEM image of ZnO nanorods grown at low temperature on top of the gold thin film is shown in Figure 1(b). The obtained ZnO nanorods were dense, vertical (in average) and relatively long. The diameter and length of the nanorods were about 170 nm and 1.56 μm, respectively.

Figures 1(c) and 1(d) show the schematic of the ZnO nanotube pH sensor and SEM image of the ZnO nanotubes, respectively. It can be seen from Figure 1(d) that obtained ZnO nanotubes are well aligned, dense and have the same dimensions as compared to ZnO nanorods (length of 1.6 μm, diameter of 170 nm and tube wall thickness of 40 nm). The stability of the (0001) and (0001̄) ZnO surfaces requires that they become less positive and negative, respectively [29]. In this chemical etching method Cl^−^ ions might be preferentially adsorbed onto the top of the nanorods to decrease the positive charge density of the (0001) ZnO surface therefore makes the (0001) ZnO surface less stable to easily etch through c-axis while chloride adsorption onto lateral walls seems to be less probable because the surface $\left(10\overset{-}{1}0\right)$ faces appear to be the most stable ZnO surface [30]. In this way the immersion time of ZnO nanorods and concentration of the etching solution were optimized taking into account the dimensions of the nanorods for the purpose of getting the nanotubes with the same dimension (length, diameter and density) without harming stable surface $\left(10\overset{-}{1}0\right)$. After the reaction, the developed sensor electrode was rinsed three times with de-ionized water to remove unnecessary chemicals and it is then ready for the construction of the pH sensor device.

### 2.2. Measurement Setup

**Figure 2.**
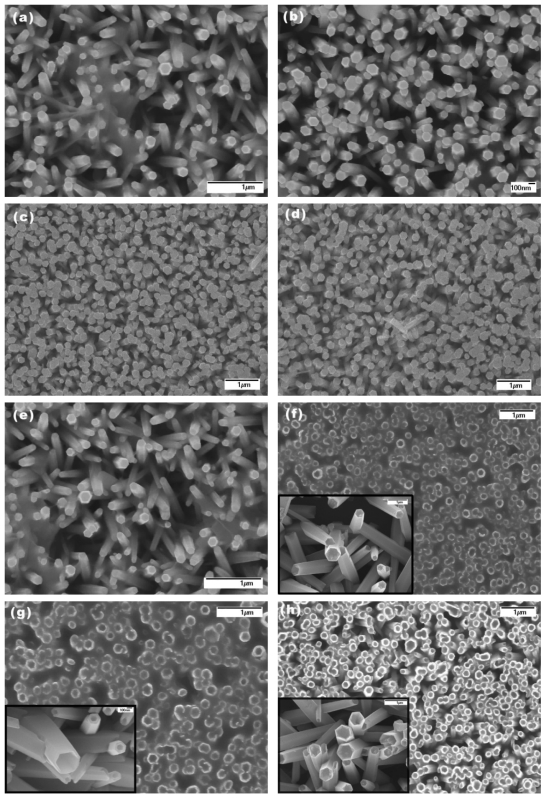

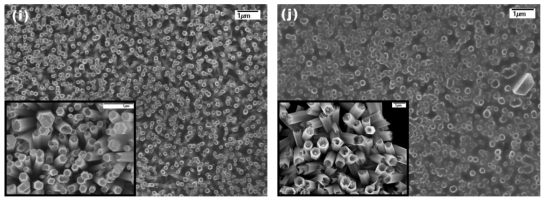
SEM images of the initial experimental results measuring ZnO nanorods/nanotubes after exposure to buffer solutions with (a) pH = 2, (b) pH = 4, (c) pH = 7, (d) pH = 8, and (e) pH = 12 for ZnO nanorods, and (f) pH = 2, (g) pH = 4, (h) pH = 7, (i) pH = 8, and (j) pH = 12 for ZnO nanotubes (the insert SEM images are the same ZnO nanotubes before exposure to corresponding pH values.)

Electrochemical studies were conducted using a two-electrode configuration consisting of ZnO nanotubes or nanorods as the working electrode and an Ag/AgCl/Cl^−^ as a reference electrode. The response of the electrochemical potential difference of the ZnO nanotubes and nanorods versus an Ag/AgCl/Cl^-^ reference electrode to the changes in buffer (purchased by Scharlau Chemie S.A) and CaCl_2_ electrolytes was measured for pH ranging from 4 to 12 using a Metrohm pH meter model 826 (Metrohm Ltd, Switzerland) at room temperature (23 ± 2 °C). The electrochemical response was observed until the equilibrium potential reached and stabilized then the electrochemical potential was measured. The real pH measurement response time of our developed sensors was less than 100 s. We have also investigated the effect of solubility and stability of the developed sensors during the experiments by constantly taking SEM images of the same samples before and after exposure to the electrolyte for each buffer pH measurement ranging from pH = 2 to pH = 12 (Figure 2 shows the SEM images for ZnO nanorods and nanotubes after each pH measurements). Some samples were dissolved at pH 2 [31]. We found that ZnO nanotubes and nanorods stay more stable at pH solutions closer to neutral pH of 7 and dissolve much faster when deviating away from pH 7. In general the effect of solubility of ZnO nanotubes and nanorods is limited to our devices because the stable potential response of each measurement was obtained within 300 s. It is very important to note that both ZnO nanotubes and nanorods are relatively stable around a neutral pH 7 and this gives these sensors much more bio-compatibility in biological fluids and species since most of the biological fluids is around pH of 7.

## 3. Results and Discussions

### 3.1. Reproducibility Test of the Developed ZnO Nanotube and Nanorod pH Sensors

**Figure 3.**
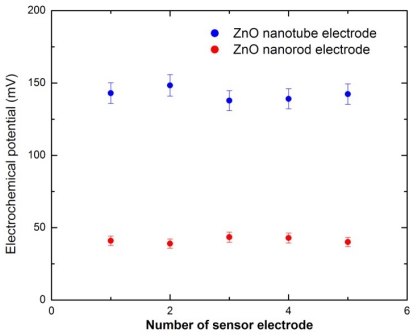
Reproducibility test of the developed ZnO nanotube and nanorod pH sensors in buffer solution at pH 6.

To obtain accurate and reusable pH sensors for application, parameters such as reproducibility, repeatability and stability were examined. Figure 3 shows the reproducibility test of the 10 independently developed ZnO nanotube and ZnO nanorod (5 ZnO nanotube electrodes and 5 ZnO nanorod electrodes) pH sensor electrodes in buffer solution at pH 6. The relative standard deviation determined from these measurements for both ZnO nanotube and nanorod pH sensor electrodes was less than 5%. Therefore, the procedure of detecting the pH of the solution is reliable.

### 3.2. Repeatability Test of the Developed ZnO Nanotube and Nanorod pH Sensors

**Figure 4.**
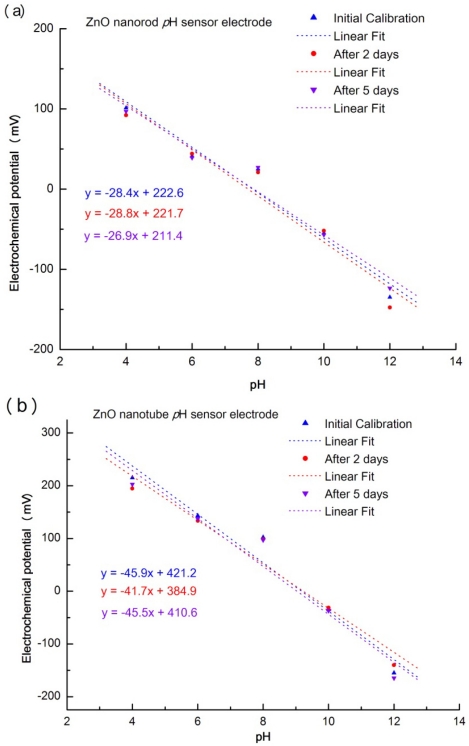
(a) Repeatability test of five ZnO nanorod pH sensor electrodes at various pH buffer solutions. (b) Repeatability test of five ZnO nanotube pH sensor electrodes at various pH buffer solutions.

Figures 4(a),(b) show good repeatability of the ZnO nanotube and nanorod pH sensor electrodes in various buffer solutions. We took 10 developed ZnO nanotube and nanorod sensor electrodes (five ZnO nanotube electrodes and five ZnO nanorod electrodes. They have the same dimensions). The responses of the sensor electrodes were separately measured in buffer solutions of pH 4, 6, 8, 10 and 12 for three experimental measurements (initial calibration, after two days and after five days). Here, the same ZnO nanotube sensor electrode or ZnO nanorod sensor electrode was used in all three measurements at a specific pH solution. The sensor electrodes were carefully washed with de-ionized water after each measurement to clean the surface of the sensor electrodes. All these results show that the ZnO nanotube or ZnO nanorod pH sensor display good reproducibility, repeatability and long term stability.

### 3.3. Comparison of the ZnO Nanotube pH Sensor and ZnO Nanorod pH Sensor

The performance of pH sensors is usually characterized by measuring the electrochemical potential of the electrodes. The most important parameter for the ion-sensitive layers is the density of surface sites that form the pH-dependent surface potential. The use of the ZnO nanotubes or nanorods as a pH sensor is based on the activity at the electrolyte-nanotube/nanorod interface, in which the H^+^ specific bonding sites residing at the ZnO surface can hydrogenate after contact with the electrolyte solution. This sites can protonate or deprotonate, leading to a surface charge and a surface potential that is dependent on the electrolyte solution pH. The Helmholtz layer is developed by the adsorption of ions or molecules on the ZnO nanotube or nanorod surfaces, through oriented dipoles, or by the formation of surface bonds between the surface and species in the solution. ZnO is an ampHoteric oxide that reacts with both strong acids and bases and displays both basic and acidic properties. The metal atoms in an ampHoteric oxide must be electropositive to give the oxygen sufficient negative charge to strip an H^+^ from a neighboring H_3_O^+^. However, the metal ion must also be electronegative enough to serve as an electron acceptor from a neighboring OH^−^, i.e., [27]:

(1) $${\text{ZnO}}_{\left(S\right)}+{\text{H}}^{+}={{\text{ZnOH}}^{+}}_{\left(S\right)}$$

The electrochemical potentiometric device used here consists of using Ag_(*s*)_|AgCl_(*s*)_|Cl^−^as a reference electrode supplied at a constant potential *E*_Ag|AgCl|Cl−_, against which we measure potential of the ZnO nanotube or nanorod redox electrode. The electrochemical device in this study can be presented as [27]:

(2) $$\text{Ag}\left|{\text{AgCl}}_{\left(S\right)}\right|{\text{KCl}}_{\left(aq,1\text{M}\right)}\vdots {\text{H}}_{2}\text{O}\left|{\text{ZnOH}}_{\left(S\right)}^{+}\right|{\text{ZnO}}_{\left(S\right)}$$

The device electromotive force (emf) is the potential difference (*E*) between the supplied potential of the ZnO redox working electrode *E*_ZnO/ZnOH_^+^ and the supplied potential of the silver/silver chloride reference electrode *E*_Ag/AgCl/Cl_^-^ [27]:

(3) $$E=E_{\text{ZnO}/{\text{ZnOH}}^{+}}-E_{\text{Ag}\left|\text{AgCl}\right|{\text{Cl}}^{-}}$$

According to Nernst equation for the equilibrium, the electrode potential can be stated [27]:

(4) $$\begin{array}{l} E_{ZnO/Zn{\text{OH}}^{+}}=E_{ZnO/ZnOH^{+}}^{0}-\left(RT/nF\right)\ln\left(a_{ZnOH^{+}}/a_{ZnO}.a_{H^{+}}\right)\\ =E_{ZnO/ZnOH^{+}}^{0}-\left(RT/nF\right)\ln\left(a_{ZnOH^{+}}/a_{ZnO}\right)-\left(RT/F\right)\ln\left(1/a_{H^{+}}\right)=E_{ZnO/ZnOH^{+}}^{0}-\left(2.303\cdot R\cdot T/F\right)pH\\ =E_{ZnO/ZnOH^{+}}^{0}+m\cdot pH \end{array}$$

where $E_{ZnO/ZnOH^{+}}^{0}$ is the standard electrode potential of the ZnO redox electrode, *R* is the gas constant (8.314 J/mol·K), *T* is the absolute temperature (298 K), *F* is the Faraday constant (96487.3415 C mol^−1^), n is the number of electrons per mole and *a*_H_^+^ is the concentration of H^+^. At room temperature (*T* = 298 K), the slope m should be:

(5) $$m=-59.1\;\text{mV}\;{\text{pH}}^{-1}$$

**Figure 5.**
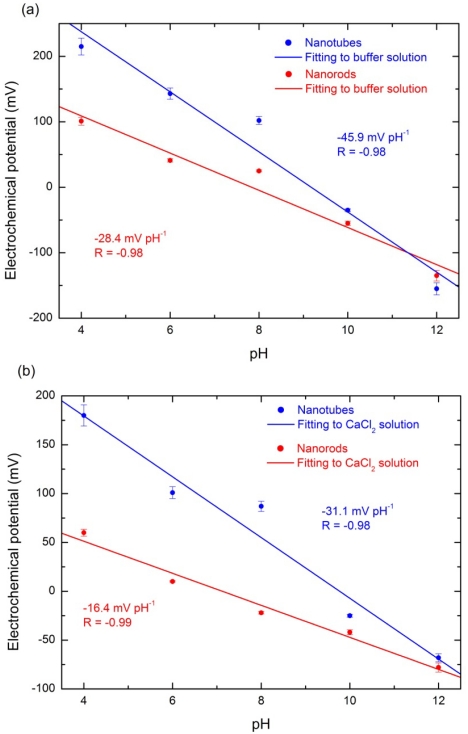
Experimental measurements of electrochemical potential vs pH comparison curves for ZnO nanorods and nanotubes immersed in (a) buffer and (b) CaCl_2_ solutions.

As shown in Figure 5(a) the potential of the ZnO nanotube or nanorod sensor electrode is linearly dependent on the pH value of buffer solutions with pH range from 4 to 12. The sensitivity of ZnO nanotube electrode is about −45. 9 mV pH^−1^ and sensitivity of the same dimension ZnO nanorod electrode is about −28.4 mV pH^−1^. The both sensitivity are lower than the theory value (−59.1 mV pH^−1^). This discrepancy may be due to the morpHology of the ZnO nanotubes and nanorods. Etching of ZnO nanotubes based on previously grown nanorods is a chemical process that may damage the surface of the ZnO nanotubes by introducing more defects or vacancies and this may affect the sensitivity of ZnO nanotubes. The most important discovery in our experiments was that we found an increased sensitivity of the ZnO nanotube pH sensor electrodes (−45.9 mV pH^−1^) in buffer solutions that is 1.6 times that of the ZnO nanorod pH sensor electrodes (−28.4 mV pH^−1^). Different chemicals are likely to exhibit different degrees of interaction with the ZnO surface. In order to investigate the ZnO nanotubes have also higher sensitivity than ZnO nanorods in different solutions we immersed the ZnO nanotube and nanorod pH sensor devices into a CaCl_2_ solution to measure the electrochemical response. Figure 5(b) shows the experimental measurements of electrochemical potential of ZnO nanotubes and nanorods in CaCl_2_ solution and it is obvious that sensitivity of ZnO nanotube pH sensor electrode (−31.1 mV pH^−1^) is about twice of that of ZnO nanorod pH sensor electrode (−16.4 mV pH^−1^) which proves that ZnO nanotube pH sensor has higher sensitivity compared to ZnO nanorod pH sensor in different solutions.

**Figure 6.**
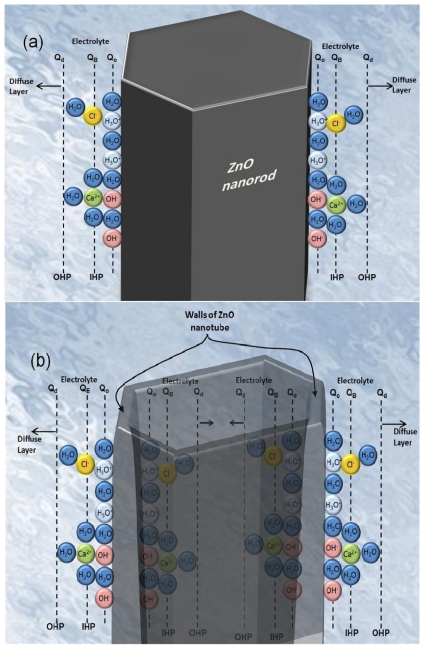
Schematic diagram showing the charge distribution at the metal oxide-electrolyte interface with surface charges in CaCl_2_ for (a) ZnO nanorods and (b) ZnO nanotubes.

The enhanced sensitivity of the ZnO nanotube pH sensor compared to ZnO nanorod pH sensor can be interpreted as a result of a larger effective surface area with higher surface-to-volume ratio [32] (Figures 6(a),(b) show the schematic diagram of the ZnO nanorod and nanotube immersed in the CaCl_2_ electrolyte showing the charge distribution at two walls of metal oxide-electrolyte interface with surface charges and ZnO nanotube has a larger effective surface). In addition, existence of a greater fraction of surface and subsurface oxygen vacancies in the ZnO nanotubes than in the nanorods and nanowires may cause enhanced performance and sensitivity in ZnO nanotubes as compared to ZnO nanorods [33].

## 4. Conclusions

In summary, we have shown for the first time that the newly developed ZnO nanotube pH sensor has higher sensitivity in different electrolytes as compared to a ZnO nanorod pH sensor with the same dimensions. Our experimental results showed that ZnO nanotubes provide sensitivity as high as twice that of identical ZnO nanorods, which can be ascribed to the fact that small dimensional ZnO nanotubes have a higher surface area and subsurface oxygen vacancies and provide a larger effective surface area with higher surface-to-volume ratio as compared to ZnO nanorods thus enables the ZnO nanotube pH sensor a higher sensitivity. A good linear electrochemical potential response was observed and our devices showed good sensitivity and reproducibility. Our results indicate that the fabrication of ZnO nanotubes can be used for pH sensor applications with improved performance. Moreover, the ZnO nanotube arrays may find potential application as a novel material for measurements of intracellular biochemical species within single living cells since nanoscale ZnO nanotube structures can miniaturize the size of the sensor in a significant way [34].

## References

[1] Wadeasa A., Nur O., Willander M. (2009). The effect of the interlayer design on the electroluminescence and electrical properties of n-ZnO nanorod/p-type blended polymer hybrid light emitting diodes. Nanotechnology.

[2] Willander M., Lozovik Y.E., Zhao Q.X., Nur O., Hu Q.H., Klason P. (2007). Excitonic effects in ZnO nanowires and hollow nanotubes. Proc. SPIE.

[3] Riaz M., Fulati A., Zhao Q.X., Nur O., Willander M., Klason P. (2008). Buckling and mechanical instability of ZnO nanorods grown on different substrates under uniaxial compression. Nanotechnology.

[4] Riaz M., Fulati A., Yang L.L., Nur O., Willander M., Klason P. (2008). Bending flexibility, kinking, and buckling characterization of ZnO nanorods/nanowires grown on different substrates by high and low temperature methods. J. Appl. Phys..

[5] Liu J.P., Guo C.X., Li C.M., Li Y.Y., Chi Q.B., Huang X.T., Liao L., Yu T. (2009). Carbon-decorated ZnO nanowire array: a novel platform for direct electrochemistry of enzymes and biosensing applications. Electrochem. Commun..

[6] Lupan O., Chai G., Chow L. (2008). Novel hydrogen gas sensor based on single ZnO nanorod. Microelectro. Eng..

[7] Umar A., Rahman M.M., Al-Hajry A., Hahn Y.-B. (2009). Highly-sensitive cholesterol biosensor based on well-crystallized flower-shaped ZnO nanostructures. Talanta.

[8] Umar A., Rahman M.M., Vaseem Mohammad., Hahn Y.-B. (2009). Ultra-sensitive cholesterol biosensor based on low-temperature grown ZnO nanoparticles. Electrochem. Commun..

[9] Umar A., Rahman M.M., Hahn Y.-B. (2009). ZnO nanorods based hydrazine sensors. J. Nanosci. Nanotechnol..

[10] Umar A., Rahman M.M., Hahn Y.-B. (2009). Ultra-sensitive hydrazine chemical sensor based on high-aspect-ratio ZnO nanowires. Talanta.

[11] Umar A., Rahman M.M., Kim S.H., Hahn Y.-B. (2008). Zinc oxide nanonail based chemical sensor for hydrazine detection. Chem. Commun..

[12] Bai X.D., Gao P.X., Wang Z.L., Wang E.G. (2003). Dual-mode mechanical resonance of individual ZnO nanobelts. Appl. Phys. Lett..

[13] Wang Z.L., Song J.H. (2007). Piezoelectric nanogenerators based on Zinc oxide nanowire arrays. Science.

[14] Zhao Q.X., Klason P., Willander M., Bergman P.J., Jiang W.L., Yang J.H. (2006). Synthesis and characterization of ZnO nanostructures grown on Si substrates. Phys. Scr..

[15] Lupan O., Chow L., Chai G., Roldan B., Naitabdi A., Schulte A., Heinrich H. (2007). Nanofabrication and characterization of ZnO nanorod arrays and branched microrods by aqueous solution route and rapid thermal processing. Mater. Sci. Eng. B..

[16] Zhao Q.X., Klason P., Willander M. (2007). Growth of ZnO nanostructures by vapor-liquid-solid method. Appl. Phys. A: Mat. Sci. Process..

[17] Kitamura K., Yatsui T., Ohtsu M. (2008). Optical and Structural Properties of ZnO Nanorods Grown on Polyimide Films. Appl. Phys. Express.

[18] Batista P.D., Mulato M. (2005). ZnO extended-gate field-effect transistors as pH sensors. Appl. Phys. Lett..

[19] Kang B.S., Ren F., Heo Y.W., Tien L.C., Norton D.P., Pearton S.J. (2005). pH measurements with single ZnO nanorods integrated with a microchannel. Appl. Phys. Lett..

[20] Al-Hilli S.M., Öst A., Strålfors P., Willander M. (2007). ZnO nanorods as an intracellular sensor for pH measurements. J. Appl. Phys..

[21] Wang J.X., Sun X.W., Huang H., Lee Y.C., Tan O.K., Yu M.B., Lo G.Q., Kwong D.L. (2007). A two-step hydrothermally grown ZnO microtube array for CO gas sensing. Appl. Phys. A.

[22] Hsueh T.J, Chang S.J., Hsu C.L., Lin Y.R., Chen I.C. (2008). ZnO nanotube ethanol gas sensors. J. Electrochem. Soc..

[23] Kong T., Chen Y., Ye Y.P., Zhang K., Wang Z.X., Wang X.P. (2009). An amperometric glucose biosensor based on the immobilization of glucose oxidase on the ZnO nanotubes. Sensor. Actuator. B.

[24] Greene L.E., Law M., Goldberger J., Kim F., Johnson J.C., Zhang Y., Saykally R.J., Yang P. (2003). Low-temperature wafer-scale production of zno nanowire arrays. Angew. Chem. Int. Ed..

[25] Vayssieres L., Keis K., Lindquist S.E., Hagfeldt A. (2001). Purpose-built anisotropic metal oxide material: 3D highly oriented microrod array of ZnO. J. Phys. Chem. B.

[26] Li Q., Kumar V., Li Y., Zhang H., Marks T.J., Chang R.P.H. (2005). Fabrication of ZnO nanorods and nanotubes in aqueous solutions. Chem. Mater..

[27] Al-Hilli S.M., Al-Mofarji R.T., Klason P., Willander M., Gutman N., Sa'ar A. (2008). Zinc oxide nanorods grown on two-dimensional macroporous periodic structures and plane Si as a pH sensor. J. Appl. Phys..

[28] Elias J., Tena-Zaera R., Wang G.Y.S., Levy-Clement C. (2008). Conversion of ZnO Nanowires into Nanotubes with Tailored Dimensions. Chem. Mater..

[29] Noguera C. (2000). Polar oxide surfaces. J. Phys. Condens. Matter..

[30] Meyer B., Marx D. (2003). Density-functional study of the structure and stability of ZnO surfaces. Phys. Rev. B..

[31] Zhou J., Xu N.S., Wang Z.L. (2006). Dissolving behavior and stability of ZnO wires in biofluids: a study on biodegradability and biocompatibility of ZnO nanostructures. Advan. Mater..

[32] Qiu Y.F., Yang S.H. (2008). Kirkendall approach to the fabrication of ultra-thin ZnO nanotubes with high resistive sensitivity to humidity. Nanotechnology.

[33] Zhang G.Q., Adachi M., Ganjil S., Nakamura A., Temmyo J., Matsui Y. (2007). Vertically aligned single-crystal ZnO nanotubes grown on γ-LiAlO_2_(100) substrate by metalorganic chemical vapor deposition. Japan. J. Appl. Phys..

[34] Asif M.H., Fulati A., Nur O., Willander M., Brännmark C., Strålfors P., Börjesson S.I., Elinder F. (2009). Functionalized zinc oxide nanorod with ionopHore-membrane coating as an intracellular Ca^2+^ selective sensor. Appl. Phys. Lett..

